# Adaptive Just-in-Time Intervention to Reduce Everyday Stress Responses: Protocol for a Randomized Controlled Trial

**DOI:** 10.2196/58985

**Published:** 2025-01-22

**Authors:** Jillian A Johnson, Matthew J Zawadzki, Martin J Sliwinski, David M Almeida, Orfeu M Buxton, David E Conroy, David Marcusson-Clavertz, Jinhyuk Kim, Robert S Stawski, Stacey B Scott, Christopher N Sciamanna, Paige A Green, Emily M Repka, Meynard John L Toledo, Nicole L Sturges, Joshua M Smyth

**Affiliations:** 1 Comprehensive Cancer Center Atrium Health Wake Forest Baptist Winston-Salem, NC United States; 2 Department of Psychological Sciences University of California Merced Merced, CA United States; 3 Department of Human Development and Family Sciences The Pennsylvania State University University Park, PA United States; 4 Department of Biobehavioral Health The Pennsylvania State University University Park, PA United States; 5 Department of Kinesiology The Pennsylvania State University University Park, PA United States; 6 Department of Psychology Linnaeus University Växjö Sweden; 7 Department of Informatics Shizuoka University Shizuoka Japan; 8 Department of Human Development and Family Studies Utah State University Logan, UT United States; 9 Department of Psychology Stony Brook University Stony Brook, NY United States; 10 Department of Medicine The Pennsylvania State University Hershey, PA United States; 11 National Cancer Institute National Institutes of Health Bethesda, MD United States; 12 Center for Healthy Aging The Pennsylvania State University University Park, PA United States; 13 Center for Economic and Social Research University of Southern California Los Angeles, CA United States; 14 Center for Survey Research The Pennsylvania State University Harrisburg, PA United States; 15 Department of Psychology The Ohio State University Columbus, OH United States

**Keywords:** stress, stress responses, stress management, just-in-time adaptive intervention, sleep, physical activity, behavior change, experimental medicine approach

## Abstract

**Background:**

Personalized approaches to behavior change to improve mental and physical health outcomes are needed. Reducing the intensity, duration, and frequency of stress responses is a mechanism for interventions to improve health behaviors. We developed an ambulatory, dynamic stress measurement approach that can identify personalized stress responses in the moments and contexts in which they occur; we propose that intervening in these stress responses as they arise (ie, just in time; JIT) will result in positive impacts on health behaviors.

**Objective:**

This study aims to (1) use an experimental medicine approach to evaluate the impact of a smartphone-delivered JIT stress management intervention on the frequency and intensity of person-specific stress responses (ie, stress reactivity, nonrecovery, and pileup); (2) evaluate the impact of the JIT intervention on the enactment of health behaviors in everyday life (physical activity and sleep); and (3) explore whether changes in stress responses mediate the interventions’ effects on health behaviors.

**Methods:**

In a 2-arm phase 2 clinical trial, we will enroll 210 adults in either a JIT stress management intervention or an active control condition. For 4 weeks, participants will complete 8 brief smartphone surveys each day and wear devices to assess sleep and physical activity. After a 1-week run-in period, participants will be randomized into the JIT intervention or an active control condition for 2 weeks. Participants in the JIT intervention will receive very brief stress management activities when reporting greater than typical stress responses, whereas control participants will receive no personalized stress management activities. Participants enrolled in both conditions will engage in self-monitoring for the entire study period and have access to a general stress management education module. Self-report outcomes will be assessed again 1 month after the intervention. We will use mixed-effects models to evaluate differences in person-specific stress responses between the intervention and control groups. We will conduct parallel analyses to evaluate whether the intervention is associated with improvement in health behavior enactment (ie, sleep and physical activity). The Pennsylvania State University Institutional Review Board approved all study procedures (STUDY00012740).

**Results:**

Initial participant recruitment for the trial was initiated on August 15, 2022, and enrollment was completed on June 9, 2023. A total of 213 participants were enrolled in this period. Data are currently being processed; analyses have not yet begun.

**Conclusions:**

We anticipate that this research will contribute to advancing stress measurement, thereby enhancing understanding of health behavior change mechanisms and, more broadly, providing a conceptual roadmap to advance JIT interventions aimed at improving stress management and health behaviors.

**Trial Registration:**

Clinicaltrials.gov NCT05502575; https://clinicaltrials.gov/study/NCT05502575

**International Registered Report Identifier (IRRID):**

DERR1-10.2196/58985

## Introduction

### Background

Broadly defined stress processes (eg, chronic stress and allostatic load) are associated with an increased risk for poor mental and physical health outcomes [1]. Traditional research on this topic tends to focus on between-person assessment examining, for example, whether those with more major life events or more chronic stressors for relatively long periods have worse health. However, chronic stress is characterized by a series of repeated acute events, each including exposure to stressors and slow and delayed recovery from stressful events [2]. Thus, to effectively characterize and intervene in stress processes, a granular measurement of acute stress is needed that uses a personalized, within-person approach. In this case, the goal is to understand an individual’s stress experience at a given moment compared to another moment (eg, their normal resting state). Such an approach captures the dynamic and temporal nature of the stress experience that occurs within an individual as they navigate their natural environment. Furthermore, this within-person approach to capturing stress experiences affords the opportunity to identify and intervene in such processes before, or at the onset of, their occurrence to attenuate the association between stress and poor health outcomes [3-5].

To help advance stress measurement approaches, we developed and refined methods for the self-report assessment needed to measure and characterize how individuals experience stress in their everyday lives in a way where each stress event can be adequately captured and studied [4,5]. We proposed that a stress event can be broken down into distinct components. Specifically, our approach focuses on assessing events that elicit immediate emotional and cognitive responses (ie, reactivity), the degree to which we adapt and recover (ie, recovery), and the temporal patterns of responding to and recovering from multiple stressors over time (ie, pileup). This assessment strategy took a within-person approach that permitted the repeated assessment of stress experiences close to their occurrence. It estimated how these stress response components (ie, reactivity, recovery, and pileup; RRP) unfold with time and in natural contexts. We demonstrated the utility of assessing these specific components of the stress responses (ie, RRPs) across 3 indicators (ie, negative affect, perseverative cognition, and subjective stress) to index “moments of risk,” where such moments are specific to a person and context [6-8]. Furthermore, we were able to link specific stress response targets to health behaviors (ie, sleep and physical activity) both within and between individuals [9,10]. Given that these stress response components and indicators are related to the enactment of health behaviors in everyday life, we contend that they may be potent targets for intervention, and we aim to test this using the experimental medicine approach [11-13].

There is growing awareness and need for better personalized and precision medicine approaches in the behavioral sciences [14]. Advances in smartphone technology (ie, apps, location, and activity) and the widespread use of mobile and sensing devices have led to the development of ecological momentary interventions that use contextual information and self-report data to match intervention content to contextual needs and deliver that content in real time [3,15]. A just-in-time (JIT) intervention framework advances this approach using rule-based algorithms to deliver the right intervention at the right time based on prespecified triggers (eg, specific variable, context, or combination of variables and context).

We suggest that our stress response assay is well suited to inform the JIT and related interventions. First, our approach is highly personalized in deriving person-specific estimates of momentary or daily risk. We have chosen to not implement static threshold–based general rules applied across all individuals; for example, with the intervention content delivered when some predetermined threshold value is exceeded (eg, a score of ≥6 on a 7-point scale) or when individuals report being in a specific location deemed risky. Although intervention delivery using this method may still benefit the recipient, it does not fully allow for adaptation to the individual with time or across changing contexts. The approach proposed in this protocol, and in some of our previous work [4,5], goes beyond using group or sample-level fixed-risk estimators, providing an adaptive and individualized framework to identify moments of maladaptive stress responses across several key indicators specific to the person. Second, we have developed, tested, and refined an intervention algorithm that uses our stress assay to process real-time information to identify and provide personalized intervention content as stress targets arise. This approach includes providing intervention content matched to general availability and the intensity and frequency of the individual’s experience. We propose that the implementation of this stress assay and the JIT intervention framework has the potential to inform approaches to individualized stress management, intervention design, and implementation science more broadly.

### Aims

This study aims to determine the effectiveness of a JIT stress management intervention, delivered via a study smartphone, on the outcomes of stress responses, sleep, and physical activity. This study will leverage our optimized stress assay to identify moments of risk and deliver empirically derived multimodal JIT intervention content in everyday life, with the aim of modifying stress response components (ie, RRPs) across multiple indicators (ie, negative affect, preservative cognition, and subjective stress) to subsequently produce positive change in daily physical activity and night sleep. Using the experimental medicine approach within the context of a phase 2 clinical trial, our primary aim is to evaluate the impact of a 2-week smartphone-delivered JIT intervention (relative to an active control consisting of self-monitoring and stress management education) on the frequency and intensity of stress responses (RRPs), as measured by (person-specific) negative affect, perseverative cognition, and subjective stress. Our secondary aim is to evaluate the impact of the JIT intervention on the enactment of health behaviors in everyday life (ie, daily physical activity and night sleep). Our third aim is to explore whether the interventions’ effects on health behaviors are mediated by changes in our target mechanism (RRPs).

## Methods

### Project Overview and Trial Design

**Figure 1:**
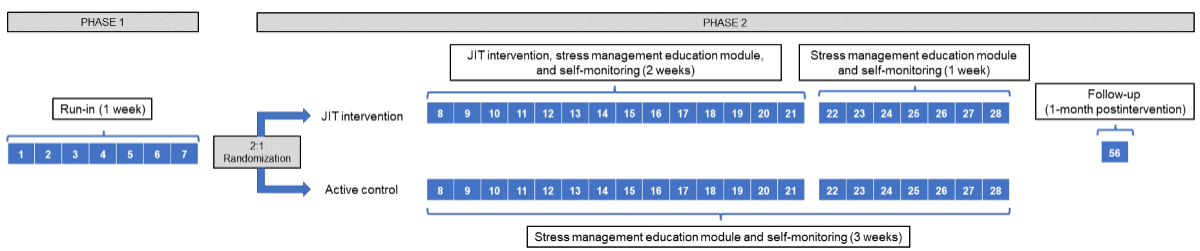
Study design. JIT: just in time.

This study is a 2-arm phase 2 clinical trial (ClinicalTrials.gov NCT05502575). The study has 2 phases (Figure 1), including a 1-week baseline eligibility “run-in” period (phase 1), followed by a 2-week randomized controlled trial (phase 2) for those eligible after phase 1, with a 1-week observational interval following the intervention.

### Ethical Considerations

The Pennsylvania State University Institutional Review Board approved all study procedures (STUDY00012740). All participants provided informed consent. All study data are deidentified and encrypted. Participants were compensated equitably for participation according to what study phases and components they completed, as detailed in the Procedures section.

### Phase 1

Phase 1 of this study serves 3 primary purposes. First, it is a data-capture period that enables us to collect individual baseline data on each participant to be used to inform the JIT intervention algorithm in phase 2. Second, it allows us to screen for participant compliance with the protocol and ensure that those enrolled in phase 2 meet minimum standards for data completeness (ie, >70%). Third, this period allows us to exclude participants who do not regularly report stress during everyday life and thus may not be impacted by our intervention.

### Phase 2

Phase 2 is the clinical trial portion of the study and will serve as the between-group test of our JIT intervention on night sleep and physical activity. This study phase will be initiated immediately following phase 1 and is 3 weeks long. Participants who are eligible for phase 2 will be randomized into one of two conditions as follows: (1) JIT intervention (ie, JIT stress management, self-monitoring, and web-based stress education module) or (2) active control (ie, self-monitoring and web-based stress education module). The first 2 weeks of phase 2 are the active intervention period, while the final week is a self-monitoring period.

### Participants

To examine the impact of our intervention on our primary outcomes without the influence of age-related stressors or increased probability of health comorbidities that could impact our measures of sleep and physical activity, our target population will be generally healthy middle-aged adults. Specifically, we will recruit English-speaking men and women aged 35 to 65 years who report good general health and are free of physical limitations from the mid-Atlantic region of the United States.

We will exclude people who report (1) residing in the same household as current or former participants (to prevent any contamination of the intervention or unblinding of condition allocation); (2) an inability to answer smartphone survey SMS text messages due to restrictions or policies in the workplace; (3) a diagnosis of a mental health condition that required a medication adjustment or hospitalization within the last 3 months; (4) being a primary caretaker for a parent, child, and family member who is severely disabled; (5) employment that requires shift work as this will result in abnormal sleep patterns; (6) a diagnosis of sleep apnea, use of a continuous positive airway pressure machine, or score above threshold on STOP-BANG; (7) the use of physician-prescribed pharmaceutical sleep aids or over-the-counter sleep aids for ≥3 days per week; (8) an inability to be physically active or who have medical contradictions for physical activity; or (9) physical exercise of ≥200 minutes per week at a moderate or vigorous intensity or ≥10 hours of walking per week as this raises the possibility of ceiling effects on activity.

### Recruitment and Screening

Participants will be recruited through advertisements posted in the local communities (eg, community centers, grocery stores, libraries, and cafés), online channels (eg, social media and study recruitment websites), and mailed flyers. Interested participants can contact the study team via phone or email and will be given a brief study overview. Participants can also access study information and preliminary web-based screening forms using a link on the recruitment materials. Individuals interested in participating will be screened to determine eligibility and scheduled for an introductory training session if eligible. All screening and scheduling will be conducted via phone using a script.

### Randomization

Randomization for this study will occur upon enrollment into phase 2 using discrete codes entered into the smartphone app by a research assistant blind to study goals. Participants will be allocated on a 2:1 ratio that favors the JIT intervention condition to provide a sample size large enough to support our planned analyses. To balance participant characteristics within the study conditions, participants will be equally randomized across 3 age categories (including those aged 35 to 44, 45 to 54, and 55 to 65 years) and by sex (male and female). Masking of the participant group identities from the training personnel, data collectors, and analysts will be used. Group assignments will be unmasked for analysis when the trial is complete and after all data have been entered.

### Sample Size

Our initial power calculations suggest that our study target for participants enrolled in phase 2 should be 210 (n=140, 66.7% assigned to the JIT intervention condition and n=70, 33.3% assigned to the active control condition). In our previous work with a similar assessment schedule, we observed compliance rates of >70% on the momentary surveys and >80% on the device wear time across 2 weeks in 95.2% (120/126) of the participants (unpublished data). Of those participants, the lowest quartile reported approximately 3 stressor events across 2 weeks. Thus, we estimate that approximately 15% of participants enrolled in phase 1 will not qualify for phase 2 due to no reported stressors or low compliance rates with study procedures. It is also likely that some participants may choose not to continue participation in phase 2 even if they are eligible due to the intensive assessment schedule. We estimate that refusal will account for 25%, and an additional 10% of those participants may either drop out during phase 1 or become ineligible. Thus, we estimate that up to 50% of the participants enrolled in phase 1 may not continue to phase 2. Therefore, we expect to recruit and enroll approximately 450 participants in phase 1 to reach our target of 210 participants in phase 2.

### Equipment

#### Study Smartphone

All study participants will be provided with an Android LG Rebel 3 smartphone. The smartphones will be preloaded with the MovisensXS app (Moviesens GmbH [16]), a secure assessment app that delivers, collects, and uploads the study smartphone surveys to a secure server. Devices will also be preloaded with the stress management intervention content. Each phone will have an active data plan and Wi-Fi capability to allow instantaneous survey uploads, intervention delivery, and access to the web-based stress education modules. Participants will only have access to the smartphone functions relevant to the study procedures, and all other smartphone capabilities will be locked.

#### Device-Assessed Sleep

Objective sleep outcomes will be collected using the Actiwatch Spectrum Plus (Philips Respironics [17]). Participants will be asked to wear the water-resistant Actiwatch on their nondominant wrist for the study’s duration, only removing the device when bathing or swimming. This device measures wrist movement time series using the digital integration method and will be configured to collect activity data at 32 Hz. At study completion, data from the Actiwatch will be processed to detect off-wrist periods and downloaded in 15-second epoch files using the Respironics Actiware software (version 6.0.9; Philips Respironics).

#### Device-Assessed Physical Activity

Participants will be asked to continuously wear a water-resistant ActivPAL4 activity monitor (PAL Technologies [18]) for the duration of the study. The ActivPAL4 monitor will be adhered to the midline of either thigh, halfway between the knee and hip, with adhesive tape. Participants will be instructed to remove the device only if bathing or swimming and will not be required to charge the device. This device uses an inclinometer and accelerometer to measure posture and activity intensity to quantify the time spent sitting, lying, standing, or stepping. Activity data will be collected at 20 Hz, with device data downloaded and processed using the PALbatch software (version 8.10.11.54; PAL Technologies).

### Procedures

#### Phase 1 (Run-In Period)

Phase 1 of this study is 1 week in duration. Participants will attend a 90-minute introductory training session with a research assistant. During this visit, participants will take part in an informed consent procedure. They will be notified that they may be invited to another longer study after they have completed the 1-week study. However, no further information about the study or eligibility criteria for participation will be provided. After informed consent is obtained, participants will participate in a study protocol training session to learn how to use the study smartphone to answer surveys. Each participant will be able to practice the surveys with a research assistant to ensure that they understand the survey items and how to operate the smartphone. Participants will also receive instruction on properly adhering and caring for the wrist-worn actigraphy device and the physical activity monitor. Finally, they will be asked to complete a demographics survey and baseline questionnaires. Each participant will receive a quick reference participant booklet that reviews relevant survey and device information.

During the 1-week study period, participants will be asked to complete 8 brief smartphone surveys per day that are delivered to the study smartphone, including 1 survey upon waking in the morning, 6 surveys delivered randomly throughout the day between 8 AM and 9 PM, and 1 survey at the end of the day before going to sleep. Participants will continuously wear the actigraphy and physical activity devices for the entire week. At the end of the 1-week study period, participants will attend a second session to return their devices and receive compensation (up to US $100). At this time, research assistants will assess survey and device data for compliance and completeness to determine whether the participant meets the criteria for eligibility into phase 2.

#### Eligibility for Phase 2

Survey data from phase 1 of the study will be used to inform the delivery of the JIT intervention in phase 2. Therefore, it is essential for participants who enter phase 2 to meet the minimum criteria for complete data on which to apply our intervention rules. For this reason, we will assess participant compliance with the study procedures and data completeness before inviting participants into phase 2. Specifically, participants who complete phase 1 will be eligible for phase 2 if they (1) return all study devices, (2) report at least 1 stressor in their surveys during the 1-week run-in period, (3) meet an acceptable rating threshold for survey compliance (ie, >70%), and (4) have valid accelerometry data for at least 5 nights during the 1-week study period. Given that these compliance checks will be conducted during a brief session, the physical activity data will not be assessed for completeness as it requires considerable time to download and review.

Once all devices are obtained and compliance checks are done, participants will be compensated according to completed study procedures. At the time of compensation, each participant will either be dismissed or invited to participate in phase 2 of the study. Those deemed eligible to participate in phase 2 will receive a brief overview of study procedures. They will participate in the consent procedure for phase 2 and a refresher training session if interested. If they are not interested, they will be dismissed.

#### Phase 2 (Clinical Trial)

Phase 2 will be initiated immediately following phase 1. The study procedures for phase 2 are similar to phase 1 (ie, 8 smartphone surveys per day and wearing study devices) but also involve randomization into either the JIT intervention condition or the active control condition. Participants will undergo another informed consent procedure and a brief review on using the study smartphones and how to wear and care for the study devices. All participants will be notified during the session that they will have access to web-based stress management education modules on the home screen of their smartphone (refer to Active Control Condition section); they may also receive additional prompts delivered to their smartphone and will be asked to follow all instructions provided to them to the best of their ability.

Given that both the participants and researchers will be blind to condition allocation, no training specific to interacting with the JIT intervention materials will be provided to participants. Importantly, no specialized training or instruction is required to participate in the intervention condition as all necessary instructions will be provided on the device when the intervention content is delivered. We adopt this approach as it may facilitate the scaling up of intervention delivery in the future (eg, by not requiring in-person or online training). After the phase 2 training session, participants will attach their study devices and continue to answer surveys delivered across the 3-week study period.

**Figure 2:**
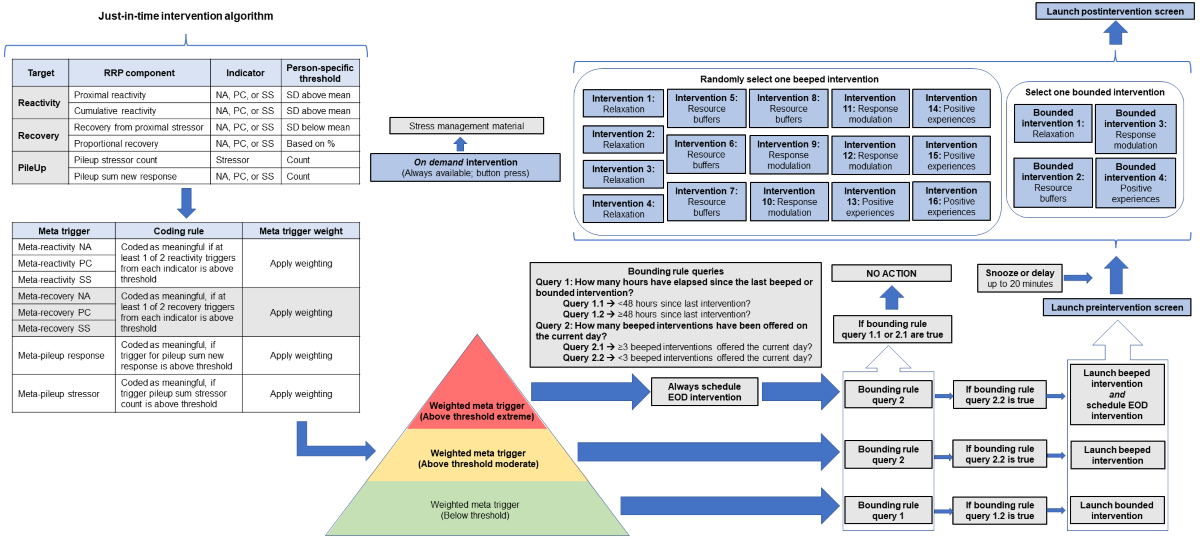
Just-in-time intervention algorithm. EOD: end of day. NA: negative affect; PC: perseverative cognition; RRP: reactivity, recovery, and pileup; SS: subjective stress.

**Figure 3:**
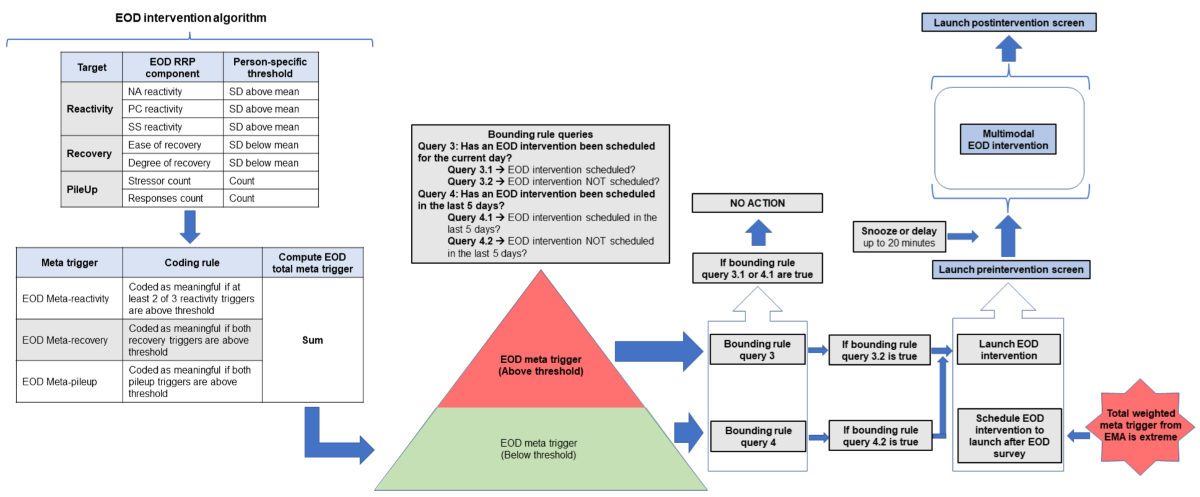
End-of-day (EOD) intervention algorithm. EMA: ecological momentary assessment; NA: negative affect; PC: perseverative cognition; RRP: reactivity, recovery, pileup; SS: subjective stress.

For the first 2 weeks of the 3-week study period, participants randomized into the JIT intervention condition will complete the 8 smartphone surveys per day, wear the 2 study devices continuously, and receive multimedia JIT intervention content to their smartphone during moments and days identified as appropriate for intervention (Figures 2 and 3). Participants randomized into the active control condition will continue to complete 8 smartphone surveys daily and continuously wear the 2 study devices. However, they will not receive any personalized stress management content. Participants in both conditions will have access to web-based stress management education information regarding the causes, consequences, and remediation of stress. This information was developed and curated by stress experts on our team (JMS, MJZ, and JAJ; ie, representing an informational standard of care) and will be available on study smartphones for the entire 3-week study period.

During the final week of the 3-week study period, the delivery of the JIT intervention content will cease (access to the web-based stress management education module will continue), and participants in both conditions will continue to engage in self-monitoring only. This final week of self-monitoring will provide information about the short-term impact of the JIT intervention and the immediate effects of tapering JIT intervention content. It will also evaluate whether any effects persist in whole or in part once the JIT intervention ceases. At the end of the 3-week study period, participants will attend a final session to return their devices and debrief about the study. A research assistant will assess participant compliance with the study procedures, and participants will receive their earned compensation of up to US $300 for phase 2.

#### Follow-Up

One month after the completion of phase 2, participants will be invited via email to complete a web-based follow-up self-report survey to assess changes in stress, sleep, and physical activity with measures capturing key study constructs. Participants will have 1 week to complete this survey to receive US $15 compensation.

### Measures

#### Demographics

At baseline, participants will provide information on the following demographic characteristics: sex, age, race, ethnicity, education level, current work status, household income, subjective social status, marital status, number of adults and children in the home, and height and weight (ie, BMI). We will also inquire about active gymnasium memberships and the use of wearables and smartphone apps for sleep and physical activity.

#### Baseline and Follow-Up Measures

The measures selected for this study are intended to capture a broad range of constructs that may impact stress and stress responses in everyday life, including personality, mood and affect, tendencies for ruminative and perseverative thinking styles, social support, and current stress. These person-level factors will help characterize our sample and may serve as potential explanatory variables for treatment response heterogeneity. We are also capturing information on current health status and preintervention and follow-up measures of self-reported sleep behaviors and sleep disturbance, physical activity behaviors, and physical activity intention or automaticity. The measures being assessed in this study and their assessment schedule are summarized in Table 1.

**Table 1:** Study measures.

Construct and scale name		Baseline	Follow-up
Health status (SF-36^a^ [19])		✓	✓
Social support (Social Support Questionnaire [20])		✓	✓
**Personality**			
	The Satisfaction with Life Scale [21]	✓	
	Life Orientation Test-revised [22]	✓	
	Big Five Inventory [23]	✓	
	Rosenberg Self-Esteem Scale [24]	✓	
**Mood and affect**			
	Positive and Negative Affect Scale [25]	✓	✓
	Toronto Alexithymia Scale [26]	✓	
**Perseverative cognition**			
	Recent Perseverative Cognitions	✓	✓
	Perseverative Thinking Questionnaire [27]	✓	
	Rumination-Reflection Questionnaire (Rumination subscale) [28]	✓	
**Stress**			
	Recent stress	✓	✓
	Current stress	✓	✓
	Stress mindset [29]	✓	✓
	Impact of Events Scale [30]	✓	✓
	Perceived Stress Scale [31]	✓	✓
**Sleep**			
	Insomnia Severity Index [32]	✓	✓
	Pittsburgh Sleep Quality Index [33]	✓	✓
**Physical activity**			
	International Physical Activity Questionnaire [34]	✓	✓
	Physical activity intentions	✓	✓
	Generic Multifaceted Automaticity Scale [35]	✓	✓
Acceptability (General acceptability)			✓

^a^SF-36: 36-Item Short Form Survey Instrument.

#### Smartphone Surveys

##### Overview

The smartphone surveys in this study capture momentary and global ratings of stressful events, subjective stress, positive and negative affect, perseverative cognitions, and health behaviors as participants go about their daily lives. There are 2 types of smartphone surveys in this study, including those that are self-launched by the participant by button press within the smartphone app (ie, morning and evening surveys) and those that are automatically delivered to the participant at semirandom intervals throughout the day (ie, beeped surveys). The self-launched surveys serve as broader retrospective assessments of these outcomes across the day. In contrast, the beeped surveys are meant to capture more proximal assessments of these outcomes in the contexts in which they occur (at the moment). The information collected in the smartphone surveys will be used to derive our indicators of stress in real time and inform the delivery of JIT intervention content (Figures 2 and 3).

##### Morning Survey

Participants will be asked to launch and complete the morning survey each day within 30 minutes of waking. This survey will assess the duration and quality of the previous night’s sleep, current stress, affect, perseverative cognitions, plans for physical activity, and expectations for the day ahead.

##### Beeped Surveys

The beeped surveys will be automatically delivered to participants at 6 random times each day between 8 AM and 9 PM during the study period and are scheduled at semirandom intervals (1 to 4 hours apart; typically 2.5 hours apart) to capture most waking hours. These brief surveys assess the participant’s current location, activities, social interactions, recent stressors, affect, perseverative cognitions, and physical activity (duration and intensity). Participants can delay (up to 15 minutes) or dismiss any survey if they cannot complete it at the indicated time.

##### Evening Survey

Participants will be asked to complete the evening survey each night before going to bed and instructed to think about their entire day (ie, from waking up) when completing the survey. This survey assesses stressful experiences and reactions to them; overall day-level affect and perseverative cognitions; physical activity (duration and intensity); consumption of caffeine, nicotine, and alcohol; use of backlit devices before going to bed; and daytime nap duration.

#### Objective Sleep

Two research staff (who have been satisfactorily trained on scoring sleep data by a sleep expert on our team) will independently score downloaded data from the sleep watch. Sleep intervals and relevant sleep outcomes will be derived using a previously validated algorithm [36]. Outcomes will include total sleep time, wake after sleep onset, sleep efficiency, and nap count or frequency and duration. Total sleep time will be calculated by the number of minutes between sleep onset and awakening. Wake after sleep onset is the total time spent being awake between sleep onset and awakening. Finally, sleep efficiency will be calculated as the proportion of actual sleep from sleep onset to awakening relative to the total sleep duration interval (%). Naps will be delineated as other sleep periods >30 minutes in length that occur outside of the main nighttime sleep period.

#### Objective Physical Activity

The physical activity data will be aggregated into 1-minute epochs. Using the sleep and wake times from the sleep data, all sleep periods will be excluded in the subsequent data aggregation process. Furthermore, any continuous sitting or standing events that are >5 consecutive hours will be classified as nonwear and will also be excluded. The remaining valid wake period will be classified into either time spent sedentary, standing, or stepping. Time spent stepping will further be classified into time spent in light physical activity or moderate to vigorous physical activity using the 100 steps per minute cut point [37]. Other relevant objective physical activity outcome variables will include step count and number of sit-to-stand transitions. The physical activity data will be further aggregated depending on the level of analysis as necessary (eg, hourly, daily, and person-level summary).

### JIT Intervention

#### General Approach

In line with the principles of the JIT intervention approach, our goal was to develop empirically supported stress management content that is delivered during particularly stressful moments. It uses a nudge framework [38] to help a person reorient to a nonstress state via improved affect and reduced perseverative and maladaptive cognitions. More broadly, each nudge aims to help build a diverse toolkit of stress management skills that can be implemented in everyday life. Furthermore, this allows us to implement a personalized approach to intervention delivery using real-time information specific to the individual at a given moment to deliver content at moments of presumptive risk or need [39].

#### Intervention Content

The development and testing of our intervention content have been described previously [40]. Briefly, we developed our intervention content within 4 broad categories: relaxation, response modulation, positive experiences, and resource buffers.

#### Relaxation

These techniques aim to shift the body from a state of elevated or negative arousal to a relaxed or neutral state. Participants could do controlled breathing, deep breathing, progressive muscle relaxation, or an object-focused meditation.

#### Response Modulation

These techniques aim to promote self-regulatory behavior, in particular, to manage emotions, promote efforts to change thoughts and associated emotions to a more positive meaning, reduce impulsive behavior, and focus attention away from emotion-eliciting situations or thoughts. Participants could perform a reappraisal task to learn how to rethink negative situations, an attentional deployment task to train them to focus on positive stimuli, a third-party observer task that asks them to imagine how an outsider would view their stress, or an affect labeling task to encourage the ability to use words to self-describe negative emotional events.

#### Positive Experiences

This category aims to facilitate engagement in activities that can induce positive mental states. Participants could imagine saying positive statements to themselves, reacting to positive imagery, engaging in a gratitude exercise, or a reminiscing task that engages them to relive a positive event in their lives.

#### Resource Buffers

This category aims to increase the quality of life at the moment to enhance function and resilience. Participants could engage in a self-affirmation task to embrace those facets of their life that are most positive, a self-efficacy task to encourage them to find moments they have successfully dealt with life events, a social comparison task that allows them to learn from others who have successfully dealt with stress in their lives, or a best possible self activity (imagining an idealized version of themselves and how they might achieve that self).

#### Intervention Types

##### Overview

The intervention content was developed to be appropriate for everyday life, including the consideration of general availability to engage with the intervention content. Therefore, we developed 2 intervention types of differing lengths and time of day availability, and all content was intended to be displayed to the participant on a smartphone. Each activity is intended for the participant to do independently, not requiring interaction with others.

##### Microinterventions

These very brief interventions (ie, microinterventions) are no more than 1 to 2 minutes in duration. This suite of microintervention content includes 16 brief videos, including 4 microinterventions across each of the 4 categories described above. For any given participant randomized into the JIT condition, when an intervention moment is identified (refer to Intervention Delivery section), 1 of the 16 microinterventions will be randomly selected from the suite of videos and presented to them on the smartphone. To maximize engagement and increase the potential for exposure to the different skills and activities, each time an intervention is delivered, it will not be placed back in the pool of available videos for potential delivery until all 16 videos have been used.

Once a microintervention is initiated, participants will receive an audible alert from the phone, and once activated, they will be notified that their stress levels appear elevated and that a recommended intervention is available to help reduce their stress. Next, they will receive a description of the activity they will engage in and how to be prepared to do it most effectively. They will then initiate the intervention video on the smartphone and follow the guided instructions. Each activity is designed so that previous training is unnecessary, but continued engagement may increase one’s enjoyment and ease in doing the activity. At the end of each intervention, we will ask the participant to consider what worked with the intervention and plan how to use it in a future moment of stress. To this end, participants must consider when they are stressed and thus engage in some self-monitoring of their behaviors. Furthermore, by encouraging participants to use the activity on their own, they may be more likely to practice and improve their skills.

##### End-of-Day Intervention

We also wanted to provide a longer, more comprehensive intervention that covered the various domains of our broader intervention approach while providing an opportunity for more focused skill-building. This 20-minute singular intervention will be delivered in the evening when people may have more time to engage in the focused activity. It comprises multimodal components that cover several broad domains and represents a progression of many of the techniques and categories noted above. The goal is to guide an individual through more extensive training to reduce stress via improved affect and reduced perseverative cognitions. It is intended to be repeated to build general skills in the broad domains it covers. Similar to what was outlined previously, participants will receive stressor-focused framing before and after the intervention.

#### Intervention Delivery

As described earlier, in our previous work, we developed a stress assay that used self-reported survey data on different stress response indicators (ie, negative affect, perseverative cognitions, and subjective stress) and several stress targets (ie, RRPs) to be able to derive person-specific means and SDs [4,5]. Using this information, we were able to establish person-specific thresholds that would indicate meaningful derivations from an individual’s norm (ie, identifying when they are stressed), as well as moments or days that were associated with poor health behavior enactment (eg, sleep and physical activity [9,10]). In this study, we aim to use this assay to identify “moments of risk” and intervene “just in time” to prevent downstream impacts on our health behaviors of interest (ie, sleep and physical activity). To provide intervention content at these moments of elevated stress while also maximizing the probability of participant engagement with the intervention content, we developed several pragmatic principles and boundaries to guide our intervention delivery. We refer to these general principles and rules as our intervention algorithms. These algorithms, when paired with the smartphone survey data in real time (ie, uploaded from the smartphone to the study server via mobile data plan), allow us to process the participant’s survey data, aggregate the information across moments and days, provide a solution (ie, trigger) for whether or not intervention content should be delivered to the participant at that moment, and subsequently deliver the content to the participant, all in near real time.

To provide us with the greatest opportunity to interfere with current or ongoing stress responses and to maximize the probability of availability, we decided that interventions would only be delivered immediately following the completion of a survey (beeped or evening). This ensures that the participant is near their study smartphone should an intervention be delivered. It also allows us to intervene in active or ongoing stress responses in the contexts in which they occur. Although this approach has some limitations, such as requiring the individual to have the mental resources to engage with the intervention during periods of elevated stress, it was chosen in an attempt to optimize intervention impact and participant engagement. This approach also provides a natural connection between this study’s intervention and survey types.

We have created 2 overlapping intervention algorithms to match intervention types (ie, microinterventions and end-of-day intervention) to survey data (ie, beeped and evening) while accounting for participant availability and burden. We developed the JIT intervention algorithm (Figure 2) to process information from the beeped surveys and trigger brief microinterventions during the day. Then, we developed the end-of-day intervention algorithm (Figure 3) to process information from the evening survey and trigger the longer end-of-day intervention. These algorithms, when combined, allow us to leverage our stress assay to detect moments of elevated stress responses in individuals at both the moment and day level, providing brief interventions in the moment or longer and more focused interventions at the end of the day, when there are generally fewer daily demands on an individual.

In broad terms, the intervention algorithms use survey data from phase 1 to derive person-specific thresholds for each participant to inform intervention delivery in phase 2. This approach highlights the importance of selecting highly adherent participants to continue to phase 2 of the study. Once enrolled in phase 2, person-specific thresholds will be continuously updated as surveys are completed and intervention content is delivered. When survey data are collected from participants throughout the day and in the evening, it will be automatically uploaded and processed in the algorithm in real time to determine whether a participant’s state indicates elevated stress levels. The algorithm will access all previous responses preceding that specific moment and the person-specific thresholds to do this. This information will then be combined with the current survey data to determine whether there is a meaningful deviation from an individual’s norm. Each meaningful score will be weighted and combined with other meaningful scores for that specific moment to derive a weighted summary risk score. Each moment’s risk level will then be evaluated based on the weighted summary risk score. Depending on this score, an intervention may be delivered to a participant at that moment, and, in some cases, a longer intervention may also be scheduled for delivery at the end of the day. This process will be repeated continuously for each participant as survey data are uploaded.

As an initial attempt to manage participant expectations and burden in this study, we incorporated a set of rules that will govern the upper and lower limits of intervention delivery (ie, ensure both a minimum and maximum frequency) and serve 2 purposes. First, our lower bound rules ensure that at least some intervention material is pushed out to each participant who is randomized into the JIT intervention condition, even in the absence of person-specific elevated stress. This includes providing a microintervention at least every 48 hours, regardless of whether elevated stress levels triggered an intervention, and an end-of-day intervention at least once every 5 days. We think this approach will keep participants engaged while providing a minimum “dose” of intervention content. We are also aware that participants in this study may experience stress below our intervention threshold (especially as their time and exposure to intervention content progresses) or that we may miss some stress responses or events with our survey schedule. Therefore, providing some minimum level of intervention content could provide the skills or resources to maintain the treatment effect, prevent future stress responses, or buffer any ongoing subthreshold stress. Second, the upper bounds of our intervention delivery will prevent us from overwhelming participants should they experience a bout of frequent or intense stress. To prevent undue participation burden related to engaging in microintervention content, we will limit microinterventions to a maximum of 3 on any given day (between 8 AM and 9 PM). We will provide the option to dismiss or delay any intervention for up to 20 minutes. We will have access to the uptake of all intervention content, including the date and time it was delivered and initiated by the participant, its duration, and the completion time.

#### Active Control Condition (Self-Monitoring and Stress Management Education)

In this study, participants enrolled in the active control condition will engage in self-monitoring for the duration of phase 2. Although there is some evidence that self-monitoring on its own might be a strategy for reducing negative affect and perseverative cognition [41], we wanted to provide a more stringent test of the “just in time” aspect of our stress management intervention. Therefore, we decided to bolster our comparison condition by providing access to a stress management education module comparable to any usual care stress management resource that an individual could access if needed. With this in mind, we developed 8 brief readings and tips that focused on stress education and the impact of stress in everyday life and adapted these materials into a web-based reading or reference module [40]. All participants enrolled in phase 2 of the study will have access to this module on the home screen of their study smartphone. If the module link is activated, they will be provided with a summary of the purpose of the module, followed by a list of 8 topics they can choose. Each module will take about 2 minutes to read and is followed by a brief, generic stress management tip that can be incorporated into everyday life. The stress management tips were derived from the same 4 categories outlined earlier, with 2 activities from each of the 4 categories included in the module. Participants will have access to this module 24 hours per day for the entirety of phase 2 and can access the readings as often as they want. User statistics will be collected each time a participant accesses the module, including the topics accessed, the number of times the module is accessed per day across the study, and a singular item about acceptability.

#### Proposed Analyses and Analytical Approach

All collected data will be subjected to a rigorous data-cleaning procedure to identify invalid data points (eg, out-of-range, implausible, and outlier variables will be scrutinized). General descriptive statistics (ie, mean, median, percentages, and SD) will be used to characterize the study sample. To evaluate the effect of the smartphone-delivered JIT intervention on RRP (specific aim 1), we will use mixed-effects models to evaluate any significant differences in RRP scores between the 2 study groups (ie, intervention group vs control group). In addition to the main effect differences between the 2 groups, we are interested in the potential cumulative effect of receiving multiple interventions across the 2-week study duration (testing a “dose” effect of the microinterventions) or whether potential benefits do not occur immediately but rather develop during the intervention period (eg, if it takes some time for the JIT intervention to lower stress responses). To evaluate this latter effect, we will test the group-by-time interaction effect in a multilevel (eg, momentary RRP nested within days, days nested within person, and person nested in group) mixed effect model. Analyses will be conducted using the SAS statistical program (version 9.4; SAS Institute Inc).

For our second aim, evaluating whether the intervention is associated with improved health behavior levels (ie, sleep and physical activity), we will use the same approach described for our first aim. That is, we will evaluate the overall treatment effect and how this effect changes with time. Unique in this evaluation, we will test these relationships using continuous outcome (ie, the amount of time spent on these health behaviors) as well as the likelihood of meeting the threshold for recommended levels of physical activity (≥150 minutes of moderate to vigorous physical activity/week) and sleep (>7 hours/night). That is, we are interested in finding out whether participants who receive the innovative JIT intervention were more likely to meet recommended levels of physical activity or sleep.

Finally, we will test whether changes in RRPs mediate the group-level effects of the intervention on health behaviors. In other words, we want to evaluate whether any benefits observed in health behaviors are due to the changes in stress responses (RRPs). All evaluations will be conducted using an intent-to-treat analysis, with imputation conducted for missing data as appropriate, and a per-protocol (completers) analysis. Given the large number of analyses planned, we will evaluate and implement appropriate methods to reduce the likelihood of false positives (eg, false discovery corrections). Theoretically informed but more exploratory analyses will be conducted to examine if there are individual difference moderators of observed effects.

## Results

Participant recruitment for the trial was initiated on August 15, 2022. Initial enrollment was completed by June 9, 2023, with 213 participants enrolled. Each of the multiple data sources (eg, ecological momentary assessment survey reports, physical activity, and sleep) is being cleaned and processed for use. Study analyses have not yet begun.

## Discussion

### Principal Findings

In this trial, we will evaluate the effectiveness of a JIT stress management intervention, delivered via a study smartphone, on the outcomes of stress responses, sleep, and physical activity. We hypothesize that those receiving the JIT intervention and standard stress management material will show better stress response processes, greater engagement in physical activity, and better sleep than those receiving only standard stress management material.

This study will provide valuable insight into the potential utility of characterizing, in a personalized manner, components of everyday stress responses and using individualized moment and day-level risk indicators to deliver JIT stress management microinterventions (relative to generic stress management educational materials coupled with intensive self-monitoring). The JIT stress management microintervention condition is predicted to enhance stress response (ie, reduced reactivity, better recovery, and less pileup) across multiple indicators (subjective stress, affect, and cognitions) relative to the control condition. In turn, it is thought that enhanced stress response processes will allow individuals to successfully engage in more positive health behaviors; namely, the group receiving the JIT intervention will exhibit more physical activity, less sedentary time, and improved sleep.

Our stress response components (RRPs) have been developed based on preliminary work and theoretical considerations; however, it is important to note that both their specific implementation (eg, the level at which momentary risk is presumed) and how they are used to trigger JIT microintervention can be readily adapted. Thus, this study is not intended to serve to finalize or concretize the specific implementation of this approach or the intervention itself (eg, the triggering algorithm and microintervention content); rather, it is meant to serve as a foundational proof-of-concept demonstration of this approach that can (and should) be adapted, refined, and tailored by others to specific samples, contexts, and research or clinical questions. It may also be fruitful to consider matching microintervention content to the nature or type of stressor experienced and the context (eg, location and social features) in which it was experienced.

This trial requires a high level of engagement by participants. Although there is the presumptive benefit of receiving stress management to enhance motivation and no side effects as might be experienced in a pharmaceutical trial, there is the requirement of actively responding to multiple brief surveys each day for a lengthy period. Clearly, this raises the possibility of self-selection, biasing the type of person who ultimately enrolls in the study. If the results of this trial indicate positive benefits for our primary outcomes, participant burden will have to be carefully considered moving forward to address issues related to generalizability and potential for dissemination.

Several other domains represent exciting future directions for this line of work to continue to refine, extend, and enhance the JIT microintervention approach we have developed. For example, developing and implementing additional stress target measurement indicators (eg, physiology and positive affect) for inclusion in the stress assay should be possible. Similarly, this work can be extended to encompass various health behaviors at different temporal scales. This stress assay can be used for intervention in “intensive” ways and integrated into more standard intervention formats (eg, face-to-face). For example, it can generate a highly reliable between-person risk indicator for risk stratification; moreover, our assay will provide risk components to allow personalized (precision) approaches to (standard) intervention. The time-varying nature of the stress assay would also allow for the development and implementation of not only JIT but also adaptive interventions, ones that “learn” what is effective for each individual and deliver what is most helpful for that individual at that moment (or stressful moments anticipated to occur in the near future).

### Conclusions

This trial evaluates the effects of a novel, personalized JIT stress management intervention on everyday stress responses and the enactment of physical activity and sleep behaviors. Overall, we hypothesize that we will see the benefits of intervention over our active control condition and hope that this stress assay and adaptive JIT intervention approach will prove a valuable assessment and intervention tool for measuring and treating stress and stress-related disorders.

## Appendix

Multimedia Appendix 1 Peer review report from the Science of Behavior Change Common Fund Program - National Institutes of Aging (National Institutes of Health, USA).

## Abbreviations

JIT: just-in-time
RRP: reactivity, recovery, and pileup

## Data Availability

The datasets generated during and analyzed during this study are not yet publicly available while data analysis is ongoing but are available from the corresponding author on reasonable request.
