# Supplementary material for: Adaptive Just-in-Time Intervention to Reduce Everyday Stress Responses: Protocol for a Randomized Controlled Trial
Source: JMIR Res Protoc. 2025 Jan 22;14:e58985. doi: 10.2196/58985 (PMC11799819; doi:10.2196/58985)
Supplement: Multimedia Appendix 1 [file resprot_v14i1e58985_app1.pdf]

1UH2AG052167-01 ALMEIDA, DAVID

INCLUSION OF MINORITIES PLAN UNACCEPTABLE

RESUME AND SUMMARY OF DISCUSSION: The proposed project, submitted in response to the NIH Science of Behavior Change initiative, develops and tests an ambulatory stress assay that can be used to personalize the implementation of stress reduction methods focused on sleep and physical activity. The project is highly innovative and potentially impactful in addressing individual-specific kinetics of ambulatory stress experiences and their impact on health behavior. Identifying at an individual level the target mechanisms of acute stress that influence health behavior and decision making fills an important gap in the existing literature and holds significant promise for developing timely and dynamic interventions. The project is guided by a precision medicine approach to stress reactivity that is manifested in within-individual manipulations and sophisticated statistical modeling. Not only does the approach hold substantial promise for developing individually-tailored interventions but it is further bolstered by the replication across ten medium to large studies within Phase 1 which bodes well for the generalizability of the findings. During discussion, however, the Committee noted several issues that detract somewhat from these strong points. For example, potential cross-contamination between two of the putative stress targets (recovery and pile-up) poses a challenge to the proposed validation, the health behaviors lack specificity and sensitivity for validating the stress targets and stress indicator variables (negative affect and perseverative cognitions) are questionable for validating the assay. More broadly, the project lacks mechanistically-driven intervention strategies, and insufficient justification is provided for the limited diversity of the validity and intervention sample in Phase 2. Although reviewers differed in weighting these issues, there was consensus that the project's precision medicine approach has promise for identifying specific ambulatory stress responses that can serve as triggers for individually-tailored interventions for promoting healthy behavior.

DESCRIPTION (provided by applicant): Stress is a documented risk factor for cardiac, metabolic, and neurological disease. How individuals respond to everyday stressors can interfere with whether they meet national guidelines for health behaviors such as exercise and sleep. The overarching goal of this project is to utilize an experimental medicine approach to develop an efficient, ecologically valid, within-person approach to measuring and intervening on the deleterious effects of everyday stress on meeting recommended levels of two health behaviors: physical activity and sleep patterns. In Phase 1, we will develop, validate and deliver a stress assay that assesses malleable components of the stress process that drive health behavior decisions and enactment as they unfold, in real-time and in individuals' natural environments. In Phase 2, we will use this assay to evaluate "just-in-time" intervention approaches that target specific stress response components at times and in contexts when they are most malleable and can positively impact health behaviors. In contrast to previous daily stress studies, we will conduct coordinated analyses in 10 intensive longitudinal datasets separating effects of stressor reactivity, recovery and pile-up on health behaviors. By replicating the results across 10 studies we will ensure identification of the most reliable and potent targets for intervention. The central hypothesis, drawn from stress theory, is that larger initial stressor reactivity, incomplete or slow recovery, and more frequent stress responses will negatively impact health behavior engagement. The rationale for the proposed research is that by identifying the strongest predictors of these health behaviors, stress interventions will be more effective in increasing physical activity and sleep to meet current guidelines (e.g. Center for Disease Control- promoted clinical recommendations). Specific aims extended from this hypothesis include: 1) Test the prediction across 10 intensive longitudinal datasets that stressor reactivity, recovery and pile-up can be identified and are more useful than traditional stress indices, 2) Test the prediction that these three components predict daily physical activity and sleep, 3) Identify characteristics of people who are either more at-risk (or resilient) to these stress interfering with health behaviors, 4) Validate these components in a novel sample, and 5) Test "just-in-time" interventions tied to times when individuals are vulnerable to the effects of stress. The research team has a history 10+

years collaboration and represents experts in theory, design, and analysis of daily stress and health behavior intervention. The approach is innovative because it tests theory- based predictions for health behaviors, will replicate effects across 10 datasets, and will develop and test novel and empirically-based “just-in-time” interventions from these results. The proposed research is significant by advancing our understanding of how stress influences the development of disease and by using interventions grounded in precision medicine to improve daily health behaviors to achieve their recommended levels.

PUBLIC HEALTH RELEVANCE: Components of the stress response in everyday life have large and pervasive effects on health behavior and downstream health outcomes. Developing a stress response assay that captures stressor reactivity, duration and pile-up that serve as targets for health behavior interventions will lead to substantial improvements in public health as well as savings in healthcare. costs.

CRITIQUE 1:

Significance: 3
Investigator(s): 2
Innovation: 4
Approach: 5
Environment: 1

Overall Impact: This application is submitted in response to the NIH Science of Behavior Change initiative and addresses an important topic of individual-specific kinetics of ambulatory stress experiences and their impact on health behavior, namely physical activity (PA) and sleep. Identifying target mechanisms of acute stress with significant impact on health behavior and decision making at an individual level presents an opportunity of high potential to fill an important gap in the current literature; existing studies provide limited information about intra-individual stress responses and, thus, the proposed research has promise for leading to timely and dynamic intervention(s). The emphasis on a precision medicine approach in stress reactivity is manifested in the project’s use of within-individual manipulations which holds substantial promise for developing individually-tailored interventions; this is highly significant. Detracting somewhat from the project’s contribution, however, are various aspects of the approach that include cross-contamination between two of the putative stress targets (recovery and pile-up) that poses a challenge to their validation, the low specificity and sensitivity of the health behaviors for validating the stress targets proposed, unclear implications for clinical outcomes and questionable stress indicator variables (NA, PC) for validating the assay.

1. Significance:

Strengths

- The emphasis on a precision medicine approach in stress reactivity by employing within-individual investigations and tailored intervention is of great significance, given the individual- and context-specific nature of stress reactivity and the scarcity of definitive data in this area.
- The proposed identification of recovery kinetics and (acute) cumulative effects of stressful events in addition to immediate stress reactivity would likely provide more salient insight into individual’s episodic stress experiences. This in turn may lead to delivering more timely and ecologically salient intervention.

Weaknesses

- Although investigating multiple aspects of stress experiences is commendable the application does not adequately operationalize the temporal distinction between “recovery (e.g., over hours/days)” and “pile-up (e.g., within a day, across adjacent days).” These concepts may be confounded psychologically and biologically and they may also vary depending upon the specific stressors involved. Even if recovery refers to “duration” and pile-up refers to “frequency” (or vice versa), stressors that evoke sustained responses (e.g., neuro hormonal, immunological, metabolic, etc.) are likely to have different dynamics than stressors that do not evoke sustained responses. This issue poses an obstacle in validating “target engagement” and “assays” as outlined in the application.
- Although physical activity (PA) and sleep hygiene are important in maintenance of general health, the levels and choice of engaging in PA and desired sleep behavior as behavioral outcomes is overly broad/generic and not adequately specific to the three stress targets. In addition, relevant disease/behavior outcome implications are lacking.
- Clinical implications or potential applications in vulnerable population are unclear.

2. Investigator(s):

Strengths

- The tandem of MPIs is highly qualified, with complementary expertise relevant to the proposed project. They have assembled a talented group of co-investigators; the team is clearly capable of carrying out the proposed research.
- The collaborative history of this team of investigators is well demonstrated.

Weaknesses

- Although the team is strong, the application does not fully justify the necessity or advantages of having a number of co-investigators spread across the country; the complexity of logistics and coordination among the Co-Is is neither fully addressed nor adequately justified.

3. Innovation:

Strengths

- A personalized medicine approach for investigating stress reactivity, the ambulatory stress assay and intervention delivery using a mobile device are all innovative.

Weaknesses

- Self-reports of negative affect (NA), perseverative cognitions (PC), PA, and sleep in relation to stress are not particularly novel.

4. Approach:

Strengths

- Investigation and validation of stress “target engagement” through coordinated analyses of ten separate datasets is efficient resource utilization. This could potentially lead to findings of great external validity and applicability.
- Rigorous analysis plans, including time-varying effect modeling, in the investigation of stress processes will likely provide valuable insight into time-sensitive stress reactivity and recovery kinetics; this information is valuable for informing timely interventions.

Weaknesses

- The application lacks clarity regarding the specific information from the ten studies in Phase 1 that is particularly pertinent to the identified stress targets, stress indicators and PA and sleep behavior.
- The procedures and inclusion/exclusion criteria for the (projected) 5,084 participants to be recruited across the ten studies are not fully spelled out in the application. The description of plans for data/study coordination and enrollment criteria for Phase 1 is lacking, although the analyses plans are reasonably well developed.
- Although the notion of "pile-up" in daily stress processes is generally reasonable, it appears that its utility/validity as a target would largely vary depending on the nature of repeated stressors (e.g., same vs. diverse stressors, locus of control, etc.). This issue is not satisfactorily addressed.
- NA and PC are proposed as indicators of stress responses, as affective and cognitive responses are hypothesized as "proximal mechanisms." This narrow approach, however, may miss other important stress response indicators of a somatic nature (e.g., fatigue, muscle tension, pain, etc.) in influencing PA or sleep, especially given the well-documented biological/physiological nature of stress responses.
- Coding of PA and sleep is based on the "binary indicators of whether the individual met guidelines for that behavior at that assessment or on that day." However, definitions to meet these guidelines are not clarified. In addition, the sensitivity of these binary indicators for PA and sleep, which are complex behaviors, is highly questionable in target engagement and valid stress assay development for predicting health behaviors that ultimately affect health outcomes. How is each individual's existing average behavioral pattern for these health behaviors of choice assessed and considered? Simply coding individuals as a "habitual exerciser" or not is not adequate.
- Plans for establishing the validity of an assay in Phase 1 are not adequately described. Demonstrating that "stress targets are malleable in response to manipulations" seems more relevant to the UH2 phase rather than the UH3 phase of the project.

5. Environment:

Strengths

- Excellent environment with necessary infrastructure and institutional support.

Weaknesses

- No major weaknesses noted.

Protections for Human Subjects:

Acceptable Risks and/or Adequate Protections

- Identified potential risks and protection plans are acceptable.

Data and Safety Monitoring Plan (Applicable for Clinical Trials Only):

Unacceptable

- Plans for DSMP within the Phase 2 part of the project (testing the intervention) are not adequately described.

Inclusion of Women, Minorities and Children:

- Sex/Gender: Distribution justified scientifically
- Race/Ethnicity: Distribution not justified scientifically
- Inclusion/Exclusion of Children under 21: Excluding ages < 21 not justified scientifically
- Exclusion of individuals of ages 18-21 years for Phase 1 is not satisfactorily justified; it appears that some "parent" studies include individuals as young as 18 years of age. Within Phase 2, insufficient justification is provided for the White-only enrollment plan and this is unacceptable.

Vertebrate Animals:

Not Applicable (No Vertebrate Animals)

Biohazards:

Not Applicable (No Biohazards)

Resource Sharing Plans:

Acceptable

Budget and Period of Support:

Recommended budget modifications or possible overlap identified:

- Requested travel cost is unusually high and not fully justified.

CRITIQUE 2:

Significance: 2
Investigator(s): 1
Innovation: 3
Approach: 5
Environment: 5

Overall Impact: The concept of developing an ambulatory stress assay that can be used to personalize the implementation of stress reduction methods targeting sleep and physical activity is innovative and potentially highly impactful. However, methodological limitations may limit the project's potential contribution; these include the lack of mechanistically-driven intervention strategies, limitation to Negative Affect and Perseverative Cognitive as indicators of the stress response and limited diversity of the validity and intervention sample.

1. Significance:

Strengths

- The provision of real time data monitoring to guide stress reduction techniques that could improve physical activity and sleep is highly innovative and could have a substantial impact on these outputs of stress.

Weaknesses

- Lack of sustained focus upon targeted mechanisms (Negative Affect and Perseverative Cognition) from Phase I to Phase II, since intervention strategies in Phase II include but are not limited to strategies that directly target those mechanisms.
- Assumption that different temporal components of the stress response (reactivity, recovery and pile-up) explain unique variance in outcomes (negative affect and perseverative cognition) is not well justified.

2. Investigator(s):

Strengths

- This is a very strong group of investigators. The PIs – Almeida and Smyth – are very experienced in this area of research, well funded and have excellent records of publication.
- The other investigators also have very strong records (Silwinski, Lanza, Buxton, Conroy & Sciamanna).
- Team is well balanced across basic and applied research, and across domains (stress and health, behavioral interventions, sleep and actigraphy).
- Includes expertise in behavioral health interventions (Smyth, Lanza, Sciamanna).
- Includes expertise in statistical modeling (Lanza.)
- Team organization is exceptionally well planned.

Weaknesses

- None noted.

3. Innovation:

Strengths

- Concept of developing an empirically derived ambulatory stress assay to be used to guide the precise moments of behavioral intervention is highly innovative.
- Statistical methods are innovative.

Weaknesses

- Selection of intervention strategies in Phase II is not innovative.

4. Approach:

Strengths

- General approach of using 10 existing data sets to create an ambulatory stress assay that is then validated in another sample (albeit small) , and then tested as a guide to intervention administration in a third sample is a major strength.
- Within person approach and related statistical modeling.
- Careful consideration of rules for what and when to intervene in Aim 5.

Weaknesses

- Selection of Negative Affect (NA) and Perseverative Cognition (PC) as the targets is justified by presenting supporting evidence of their linkages with health behaviors but insufficient consideration is given to alternative targets that may be even more potent, such as positive

affect, impulse control and/or habitual responding. Also, NA and PC are likely to be highly correlated and may not capture the full stress response across all individuals.

- Brief reference is made to testing links between Negative Affect and Perseverative Cognition on the one hand and cortisol on the other hand (as a type of validation of NA and PC as valid markers of the stress response) – but how this will be done is not clarified.
- The measure of stress exposure (perceived severity and subjective distress) overlaps with the measure of stress response (negative affect and perseverative cognition), since negative affect most likely influences, and will be highly correlated with, perceived severity and subjective distress.
- Ambulatory measures of sleep (i.e., actigraphy) have limited validity given that movement per se is inadequate as a measure (although improvements are occurring with more recent technology); this may tarnish modeling of sleep behaviors from the prior ten studies although it is unclear how sleep and activity were monitored in those studies (self-report versus actigraphy).
- The interventions chosen are not well justified; and only some are directly tied to the target of engagement (i.e., negative affect and perseverative cognition). The interventions comprise a large list of many strategies found to reduce stress. Identification of which strategies most directly target negative affect or perseverative cognition is missing.
- Selections of which strategies are applied when negative affectivity emerges versus when perseverative cognitions emerge, and variations based on the phase of the stress response, are also not well justified.
- Decision to implement an intervention based on momentary assessment of a stressor and elevations in NA or PC may result in multiple and repeated interventions.
- Will only include a control group in Aim 5 should there be an effect of testing in the validity sample; without a control group the effects of the interventions in Phase 2 cannot be attributed to the intervention.

5. Environment:

Strengths

- Excellent scientific resources.

Weaknesses

- The validity sample of Phase 1 and Intervention sample of Phase 2 will be recruited from Centre County Pennsylvania; limited diversity of the sample is problematic, and in addition it differs substantially from the diversity of the sample on which the ambulatory stress assay is developed.

Protections for Human Subjects:

Acceptable Risks and/or Adequate Protections

Data and Safety Monitoring Plan (Applicable for Clinical Trials Only):

Not Applicable (No Clinical Trials)

Inclusion of Women, Minorities and Children:

- Sex/Gender: Distribution justified scientifically

- Race/Ethnicity: Distribution not justified scientifically
- Inclusion/Exclusion of Children under 21: Excluding ages < 21 justified scientifically
- Sample for Phase 1 analysis of existing data sets represents a wide distribution of race and ethnicity. However, the validity sample of Phase 1I and the sample for Phase 2 is predominantly White - this is not scientifically justifiable.

Vertebrate Animals:

Not Applicable (No Vertebrate Animals)

Biohazards:

Not Applicable (No Biohazards)

Budget and Period of Support:

Recommend as Requested

CRITIQUE 3:

Significance: 2
Investigator(s): 1
Innovation: 2
Approach: 1
Environment: 1

Overall Impact: This is a highly innovative project. The within-subject focus is novel and supported by appropriate and innovative statistical models. The replication across 10 medium to large studies adds to the generalizability of the findings. The potential for high impact is considerable.

1. Significance:

Strengths

- The focus on within-subject assessment of stress is highly significant as between-subject approaches are limited in their applicability to individualized interventions.

Weaknesses

- No major weaknesses noted.

2. Investigator(s):

Strengths

- Dr. Almeida is a leading researcher on within-subject change and health. He has assembled a strong team.

Weaknesses

- No major weaknesses noted.

3. Innovation:

Strengths

- The focus on a within-subject approach is a major strength.
- Examination of recovery is also a plus.

Weaknesses

- No major weaknesses noted.

4. Approach:

Strengths

- Consistent with the within-subject focus, the statistical approach of using time-varying effect modeling (TVEM) is a strength.
- The use of 10 large data sets.

Weaknesses

- No major weaknesses noted.

5. Environment:

Strengths

- Penn State is a leading center for advanced methodology. Thus this is a major strength.

Weaknesses

- No major weaknesses noted.

Protections for Human Subjects:

Acceptable Risks and/or Adequate Protections

Data and Safety Monitoring Plan (Applicable for Clinical Trials Only):

Not Applicable (No Clinical Trials)

Inclusion of Women, Minorities and Children:

- Sex/Gender: Distribution justified scientifically
- Race/Ethnicity: Distribution justified scientifically
- Inclusion/Exclusion of Children under 21: Excluding ages < 21 justified scientifically

Vertebrate Animals:

Not Applicable (No Vertebrate Animals)

Biohazards:

Not Applicable (No Biohazards)
